# The nexus between corporate governance, risk taking, and growth

**DOI:** 10.1371/journal.pone.0228371

**Published:** 2020-02-04

**Authors:** Alin Marius Andries, Daniela Balutel, Iulian Ihnatov, Silviu Gabriel Ursu

**Affiliations:** 1 Faculty of Economics and Business Administration, Alexandru Ioan Cuza University of Iasi, Iasi, Romania; 2 Institute for Economic Forecasting, Romanian Academy, Bucharest, Romania; 3 Laboratoire d’Economie d’Orleans, Orleans, France; The Bucharest University of Economic Studies, ROMANIA

## Abstract

In this study we assess the impact of corporate governance on the risk investment behavior of firms and its implications on firms’ growth rate. Using a sample of non-financial companies from 10 countries over a period leading to the recent global financial crisis, we documented that the corporate governance has a nonlinear (inverted U-shape) impact on the companies’ investment risk, meaning that the investment risk is increasing up to a level of corporate governance of 0.61 (as measured by our comprehensive index), while at higher levels of corporate governance the investment risk is decreasing. For the models of sales growth and assets growth it is shown that predicted investment risk has a positive effect on firms’ growth measures. Moreover, the two growth models are not moving independently and a shock to one of the growth measures (sales or assets) affects the other growth measure in the same direction. Additionally, we evaluated the effect of financial crisis on both the growth measures and the risk measure. The effect of financial crisis was captured in both measures in 2009, with higher impact on the growth of sales.

## 1. Introduction

The importance of corporate governance has lately attracted much attention from academic researchers, practitioners, and policymakers, mostly due to the lack of its effectiveness as highlighted by the Global Financial Crisis (GFC). The corporate governance system not just failed to reduce asymmetric information, control managerial opportunism and redirect management toward optimal behavior, but had instead incentivized corporations to create and take excessive risks for short-term profit maximization [1, 2, 3, 4].

Given the importance of effective corporate governance, our research objective is to investigate to what extent the quality of internal corporate governance affects risk taking and growth of non-financial companies from several countries before and during the GFC.

Our paper is a part of an extended literature drawing from agency theories that examines the impact at firm level of different corporate governance variables, such as managerial incentives and characteristics, creditor rights, investor protection, cultural issues, corporate governance reform or the board of directors’ characteristics. The board of directors is an essential governance entity that alleviates the agency problem between shareholders and executives: it hires or dismisses the top management, designs the compensation schemes, ratifies the most financial decisions, and, therefore, it influences corporate risk taking [5, 6].

Managers are usually risk averse since their policy decisions are normally conservative compared to a well-diversified shareholder [7]. They will take less risky projects and, sometimes, even avoid risky projects with positive net present value [8, 9, 10, 11, 12]. Some characteristics of the management, such as the CEO social capital, may increase the riskiness of firm investment and financial policies. These policies channel the effects of social capital on aggregate corporate risk-taking [13]. Informal institutions such as culture matter in corporate decisions, even when sophisticated professional managers make those decisions in a globalized environment [14].

Our paper is one of the first to study the impact of the overall index of firm-level corporate governance on investment risk, and, further, on corporate growth. Previous governance studies are based on a narrow measure of governance and show how risky choices are affected by the ownership structure, compensation scheme and corporate cash flows that make insiders to divert for their personal benefits. Failure to control for other aspects of governance leads to a biased interpretation of the effect of the overall corporate governance practices. Compared to previous studies, our research contributes to the literature by employing a global corporate governance index that incorporates 41 minimally acceptable governance standards.

Our work is motivated by the fact that, with the exception of a few studies, much of the research is mainly based on country-level analyses of investor protection and financing or ownership structure and risk. Adding the firm level variation in this analysis provides more information about the micro-foundations that link investor protection to corporate risk. Along with the firm level variation, this paper exploits the cross-country variation. In particular, whilst there is a possibility that U.S. and non-U.S. companies might have the same level of corporate governance, our analysis exploits the differences in characteristics present at the country-level, respectively the rule of law and the strength of investor protection. Additionally, a cross-country study is relevant, taking into consideration that variation in corporate governance and investor protection is possible to be greater and more exogenous than the within countries variation.

Consequently, the empirical analysis is based on U.S. and Non-U.S. firm-level data from ten countries that spans over the period between 2004 and 2009. The rationale behind the choice of this time span is twofold. First, the span of the data given the topic of the paper reflects the behavior of the firms at a more global perspective leading into the financial crisis. In particular, we wanted to understand the relationship between governance and risk prior and during the GFC, knowing that firm governance behavior was different before crisis and post crisis. Second, the data availability is limited. Consequently, we have chosen a sufficiently large window of data prior to the financial crisis, conditioning that the data on all firms was available starting with 2004. The proposed period of analysis allows us to compute a risk measure for the whole window leading into crisis (knowing that the behavior of firm governance was different prior and during the crisis compared to the post crisis period—e.g. see [15]; and [16]).

Additionally, we constructed a time varying risk measure, which allows us to quantify the effect of the crisis both on our risk measure and on our two growth measures. Similarly to [17] we want to assess the real impacts of the 2008–2009 crisis on firm-level performance. Consequently, we generated crisis dummies for 2008 and 2009 and added them to our benchmark model specification to test if the financial crisis had a different effect on both governance and growth measures.

The results on financial crisis are mixed: the financial crisis affected only the sales growth along with the risk measure, but the effect was significant only in 2009. This delayed effect of the financial crisis on the firm growth is justified: the financial crisis was transmitted instantaneously at financial institutions level, but not at non-financial firm level.

Additionally, the non-linear effect of the Global Index of Corporate Governance was never exploited in the literature and we view this identification mechanism of the risk as a supplementary contribution. Our results reveal an inverted U-shaped relationship between the risk measure and the Global Index of Corporate Governance. This result is robust to other risk measures that capture the effect of relative distances of return on assets (ROA) from country specific, industry specific and country and industry specific averages. Using an identification mechanism in two stages, the empirical analysis tests confirm that the levels of corporate governance and investment risk are positively associated up to a certain threshold, stronger internal corporate governance and investor protection lead to higher firm-level riskiness in investment, which in turn, is positively associated with company growth. These results are partially in line with [11] that also find a positive linkage between investor protection and risk, but did not consider the non-linear effect, and further suggest that the riskier investment strategy determines a higher firm value that induces higher economic growth rate. As a robustness check, we have used two alternative firm’s growth measures (Total Assets and Sales Growth). We have also checked if the alternative growth measures were moving independent of each other. [18] show that there is a link between different types of firm’s growth measures, including the growth in sales and in assets. Consequently, we investigate how these two growth measures of interest are connected via the idiosyncratic errors of the models that are used to predict the two growth measures. We have used a Seemingly Unrelated Regression (SUR) model on the two growth models to show if a shock on a growth measure affects the other growth measure in the same direction. This additional finding suggests a strong dependence between the growth measures.

Furthermore, by using the time varying risk measure (based on a three-year rolling window standard deviation) we also exploit the time varying information from our control variables, and we are able to apply a panel data estimator. The panel data results are robust with our previous estimations that are based on the single-window firm-level risk measure (for the entire 2004–2009 period).

The reminder of this paper is organized as follows. The next section briefly reviews the literature. The third section explains the data sample, defines the variables used in the analysis, specifies the model and notes the estimation procedure. Section 4 describes the data, discusses the results on the relation between governance, risk taking and firm growth. The final section concludes with a summary.

## 2. Related literature

The corporate governance environment is changing continuously. Studies on corporate governance reform show two opposing views on the effect of reform on corporate risk-taking.

A positive association is motivated by the following findings. [11] Show that corporate risk-taking is higher in firms operating in better governed environments. Also, lower investor protection allows dominant insiders with low levels of cash flows rights to manipulate corporate resources for private interests [19, 20]. In order to protect their benefits, which are associated with diverting corporate resources, insiders will be conservative in corporate investment. Second, in countries with poor investor protection, due to the lack of shareholders diversification that uses a pyramidal ownership structure, and due to their high exposure, insiders will avoid risky investments as there are fewer corporate resources to manipulate and it is easier to detect if they try to act for their personal benefits [20, 21]. However, insiders can consider a risky project only if its expected outcomes are high enough to compensate for the lower level of diversion [11]. Third, managers that want to protect their career will usually avoid taking risky projects, even these are firm value enhancing unless they are forced by well-diversified and risk-loving large shareholders [11].

The presence of a negative relationship between investor protection and corporate risk is justified by other findings. [22] employ twelve measures that capture corporate risk-taking and show that companies in common-law countries, with stronger protection of property rights, and those in market-based financial systems take less risk. An environment with strong creditor rights stimulates the manager decision to diversify and reduce the risks [23, 24]. Therefore, in a country with weaker market-based monitoring, a substitutive effect of regulatory reform could alter insiders’ utility trade-off to pursue corporate risk-taking [25]. Other authors argue that the risk taking is discouraged by the increased personal liability of corporate insiders after the reform [26, 27]. Also, along with improved investor protection concerns regarding the expropriation of managers are diminished [28] and there will not be necessary to have a concentrated ownership to monitor managers [29]. As the number of dominant shareholders decreases, managers will have the freedom to implement a conservative investment policy. Another argument refers to a “tunneling distortion”, which suggests that in environments with poor investor protection, dominant insiders that control the company in a pyramidal way may be very common [19, 20]. In this type of structure, insiders will have the incentive to take risks in companies where their cash flow rights are low and to do the opposite in companies where their cash flows rights are high.

Those who control the firm choose a firm’s governance system in order to maximize their welfare. Investments in corporate governance reduce the controlling shareholders’ ability to extract private benefits from the firm [30]. According to a survey conducted [31] the majority of investors are willing to pay a premium for companies with high governance standards. Good corporate governance will improve companies’ confidence and investors will have the incentive to invest more and to gain more. Investing more in a company, even if could be risky, will lead to corporate growth, but will also improve the performance of the companies, industries and, in turn, the country’s economy [19]. This might be a good reason for a country to improve investor protection and to help companies to improve their internal corporate governance, especially under adverse economic conditions [32]. A common issue related to corporate governance is the problem of investor expropriation, sometimes also referred to as self-dealing. Specifically, those who control a corporation, whether they are managers, controlling shareholders, or both, can use their power to divert corporate wealth to themselves rather than sharing it with the other investors [33]. Modern corporate finance theory focuses on the ability of corporate insiders to divert corporate wealth to themselves [34]. Insiders may use corporate assets for their personal benefits. In order to protect their private benefits, sometimes insiders choose to apply a conservative behavior towards corporate investments. The more important the private interests are the more risk averse the insiders will be in the decision-making process regarding investment. In this situation insiders will avoid risky projects that may affect firm’s growth.

Considering these mixed theoretical and empirical findings about the impact of corporate governance on risk at firm level, we can infer that potentially these results are driven by the possible misspecification of the functional form that links corporate governance to risk taking.

In particular, we found that some researchers identified a nonlinear relationship between corporate financial performance and corporate governance [35]. These financial performance indicators are also linked with the risk measures proposed in the analysis. Therefore, we also want to test if the corporate governance index has a nonlinear effect on firm’s risk. A study on Italian firms [36] also shows a significant non-monotonic relationship between the risk-taking and ownership structure. Consequently, a first hypothesis of interest (H1) refers to functional form that links the corporate governance to firm’s risk and tests if the relationship is monotone or not.

A second point of interest for our analysis is the relationship between corporate risk taking and firm performance. [37] formalized a theoretical relationship between risk and growth, but more specific to our question of interest is the potential link that corporate risk-taking has on improving the efficiencies in the utilization of assets with effect on profitability of the companies on returns, and firm growth [7, 38, 11, 39]. A direct positive link between risk-taking and both company asset and sales growth as well as the relation between ownership and risk-taking is found in [11].

Consequently, our second testable hypothesis (H2) links the investing risk taking to the growth on asset and sales measures both in an independent and a joint way.

## 3. Data and methodology

### 3.1. Sample and data source

Our original sample includes data on 1039 non-financial listed companies with annual revenue more than 50 million dollars and with regular employees more than 500, with diversified ownership and strong governance practices, over the period between 2004 and 2009. The companies from the sample activate in 77 industry sectors, according to two-digits NACE code, and come from the following countries: Austria, Australia, Belgium, Denmark, Finland, France, Germany, Spain, Switzerland and U.S. We exclude financial institutions, such as banks and insurance companies, so to reduce the impact of the heterogeneity associated to performance and risk-taking behaviors of different industries. The dataset uses company balance-sheet information from Bureau van Dijk—Orbis Database and information on investor protection and governance indicators from the World Bank. Financial data is further merged with the firm-level corporate governance index from [40] based on the SEDOL code for European companies, and the CUSIP code for U.S. companies. We complied with the terms of service for the databases used in this study.

The time span of the analysis is limited to the period between 2004 and 2009 as the corporate governance index was computed [40] for the period 2002–2009 for US companies and 2004–2009 for non-US companies. The data covers a sufficiently large window of data prior to the financial crisis in order to understand how the corporate governance prior to the crisis affects the firm’s risk. Furthermore, the working sample is reduced to a number of observations between 759 and 823, depending on the variables used in different specifications of the model. For example, leverage data is available for only 855 companies and the governance measure is not observed in some cases. However, this data may be viewed missing at random, as the average on the outcome is not affected (e.g., the mean comparison tests for sales, total assets and risk measures didn’t reveal statistically significant differences between the original sample (1039 observations) and the working sample (about 760 to 830 observations)). We deal with the outliers by winsorizing the variables at 5%.

### 3.2. Description of variables

#### 3.2.1. Measurement of firms’ risk

Following previous studies, see e.g [11], [39], [41] and [42], the dependent variable is represented by the standard deviation of return on assets for each firm over the entire period (2004–2009). Volatility of returns is a standard proxy for risk in the financial economics literature [43]. The measure of firm`s risk, RISK1, is based on the variation in corporate earnings (EBITDA) divided by total assets. The deviation of each company’s EBITDA/ Total Assets is computed from the sample average for each of the six consecutive years of firm observations during the period 2004–2009. The standard deviation of this measure is then considered as the value of risk.

$${RISK1}_{i}=\sqrt{\frac{1}{n-1}\sum_{t=2004}^{2009}{\left({ROA}_{it}-\frac{1}{n}\sum_{t=2004}^{2009}{ROA}_{it})\right)}^{2},\;}n=6$$

In addition, we compute several other measures of risk based on standard deviations of the returns on assets from their means at country and industry level, as follows:

1. country means (${{\text{ROA}}^{c}}_{it}={ROA}_{itc}-\frac{1}{Nc}\sum_{c=1}^{Nc}{ROA}_{itc}$), where *i* represents the firm, *c* represents a specific country in the sample and *N*c is the number of countries,
2. industry means (${{\text{ROA}}_{it}}^{I}={\;ROA}_{itI}-\frac{1}{Nind}\sum_{I=1}^{Nind}{\;ROA}_{itI})$, where *I* represents the industry at 1 digit SIC code and *Nind* represents the number of industries at 1 digit SIC code.
3. country and industry mean (${{\text{ROA}}_{it}}^{cI}={ROA}_{itcI}-\frac{1}{Nc∙Nind}\sum_{c=1}^{Nc}\sum_{I=1}^{Nind}{ROA}_{itcI})$.

We create these additional variables to check the robustness of our results to alternative risks measures that capture the effect of relative distances of ROA from specific averages.

We also constructed a time varying risk measure (rolling window standard deviation of ROA) by using a three-year rolling window.

$$ROLLING\;SD=\sqrt{\frac{1}{n-1}\sum_{j=t}^{t+n}{\left({ROA}_{ij}-\frac{1}{n}\sum_{j=t}^{t+n}{ROA}_{ij})\right)}^{2},\;}$$

*n* = 3, j = 2004, 2005, 2006 and 2007.

This time varying risk measure allowed to use the time varying information from the data an additional panel data specification for the risk model is estimated.

#### 3.2.2. Governance variables

*Firm-level corporate governance index*. In order to measure the corporate governance, we are using the firm level Corporate Governance Global Index (GOV) index developed [40]. The composite index comprises 41 common firm-level governance attributes for U.S and non-U.S. companies. The index consists of attributes that cover four large subcategories: Board (24 attributes), Audit (3 attributes), Anti-takeover provisions (6 attributes), and Compensation and Ownership (8 attributes). Board attributes refer to the aspects of the board of directors like board independence, structure of committees, size, transparency, and how the board of directors conducts its activity. The audit subcategory includes elements on the independence of the audit committee and the role of auditors. Anti-takeover provisions refer to dual-class structure, role of shareholders, poison pills, and blank check preferred. Compensation and ownership are related to executive and director compensation on issues linked to options, stock ownership and loans, and on how compensation is established and monitored.

For each attribute, Institutional Shareholder Services (ISS) evaluates whether a firm meets a threshold level of implementation of the attribute and considers that the firm has that attribute if it meets the threshold. The index assigns a value of one to a governance attribute if the company meets the threshold level and zero otherwise. Similar to [40], we express our index as a percentage. If a firm satisfies all 41 governance attributes, its Governance index would be equal to 100%. If an attribute is missing, the attribute is eliminated and the value of the index represents the percentage of non-missing attributes that the firm satisfies.

**Fig 1:**
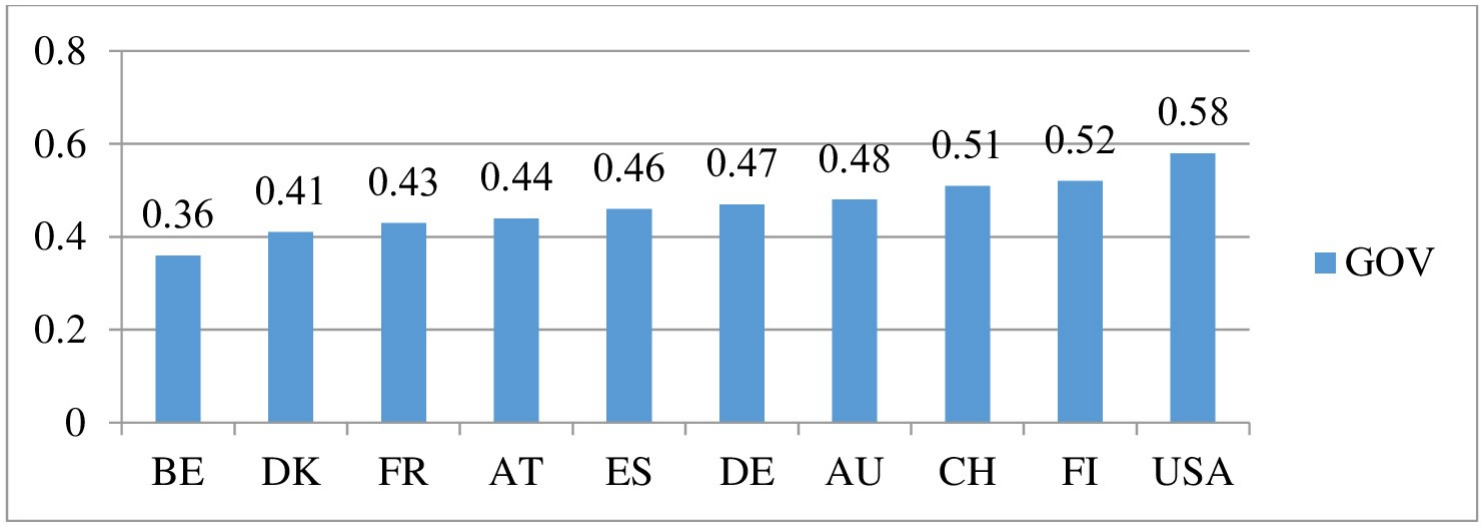
Corporate governance index by country. Source: Authors’ calculation.

Fig 1 shows the average of the firm-level corporate governance index for each country from our sample. As seen, the highest value of corporate governance index (0.58) corresponds to the United States, followed by Finland (0.52) and Switzerland (0.51). The rest of the countries have an index below 0.5, with the lowest value (0.36) attributed to Belgium.

*Country-level governance indicators*. The level of investor protection at country level can be measured in different ways. At the country level, we use two indices that measure the strength of investor protection index and the rule of law. The index of the strength of investor protection is an average of three indices (the extent of disclosure index, the extent of director liability index, and the ease of shareholder suit index); it ranges from 0 (little, to no investor protection) to 10 (greater investor protection). The index of the rule of law captures perceptions of the extent to which agents have confidence in and abide by the rules of society, in particular, of the quality of contract enforcement, property rights, police, courts, as well as the likelihood of crime and violence. This index takes values from zero to one, with higher values corresponding to a better rule of law. The source of the data is the Word Bank.

#### 3.2.3. Measuring growth

Firm-level growth is measured as the average growth of total assets or sales, over the sample period, respectively 2004–2009. First of all, we measure firm-level growth in assets for each year over the sample period, as the ratio of the total assets on year *T*_*n*_ divided by total assets on year *T*_(*n*−1)_, Secondly, we compute the average growth rate for each company. We use the same approach to compute the sales growth at firm level.

For robustness check, in a panel data analyses we have used sales and total assets growth from one financial period to another.

#### 3.2.4. Control variables

To control for differences in companies’ characteristics across the sample, we employ the following firm-level control variables: *Firm size*, *Return on assets*, *Leverage* and *Company independence*. Initial firm size of a company is defined as the natural logarithm of total assets. The firm size may influence the company behavior, as larger companies might be more risk averse when the business is stable and the returns have low volatility [11] and also, they have the ability to diversify risk due to product lines. Return on assets is used as a proxy for firm earnings, justified by the fact that sometimes this variable refers to return on investment and in general the risk can be associated with higher profitability. Return on assets is computed as the ratio of the EBITA to Total Assets. Leverage, defined as the ratio of long-term debt to total assets, serves as a proxy for the investment risk of the firm, as well as an important instrument used by firms to grow. All three variables are measured in 2004, at the beginning of the observation period. [44] and [45] showed a strong and positive relationship between leverage and the volatility of ROA and stock returns. [46], on the other hand, finds that the leverage has no impact on the return volatility for U.S. companies.

To characterize the degree of independence of a corporation with respect to its shareholders, following [11] and [47], we have used the Independence Indicator included in the Bureau van Dijk (BvD) Orbis Database. Ownership concentration influences the risk-taking behavior of companies. [48] found out that dominant owners of Brazilian firms tend to select riskier projects when pursuing self-interest goals. These excess voting rights of the largest shareholders increase their incentive to extract private benefits of control at the expense of the minority shareholders’ wealth [49]. Family control is positively associated with the firm’s idiosyncratic risk [50, 51]. *Company independence* is a dummy variable that has a value of one if the company is rated with indicator A (referring to any company with known recorded shareholders, none holding more than 25% of direct or total ownership), and a value of zero, which is attached to any company rated with B, C, D and U (with a known number of shareholders that have more than 25% of direct or total ownership).

S1 Appendix includes the definitions of all variables in the analysis.

### 3.3. The model

To assess what is the channel that links firm’s governance to firm’s growth we use an identification strategy similar to [11] and [45]. However, we extended their identification strategy in several ways.

First, we test the nonlinearity effect that corporate governance has on a firm’s investment risk. This hypothesis being an important pillar for our identification as corporate governance may not have a linear effect on risk. This nonlinearity may be driven by the fact that an increase in regulatory compliance may affect negatively the efficiency of management for firms by increasing their reaction time and costs associated to this compliance, see [52]. To do this exercise we constructed a more comprehensive measure of corporate governance. Consequently, we consider the model that links corporate governance to the firm’s risk firstly as an independent model, and secondly we link this model to the firm’s growth. As a result, we test also what is the effect of investment risk a firm takes on its growth.

The proposed identification strategy consequently is different than the one used in the above-named reference. Additionally, we used a multilevel analysis at country and firm level for a period that leads to financial crises. Therefore, we can also test how corporate governance affects firm’s investment risk before and during the crises, hypothesis that is verified via an interaction between governance and crises dummy (S8 Appendix).

There are several reasons to perform our analysis at the cross-sectional level using the first year we observe the governance measure for US and Non-US companies (2004) as the benchmark year. First, the measure of risk that covers the full period is computed as a deviation from the company’s return on assets, which makes it fixed over the sample period. Second, the outcome measures were computed as yearly average growth rates over the window period (to match the aggregation of information that was used on the computation of the risk measure); therefore, they are also not varying over time. Moreover, the firm-level information used as control variables is very persistent, as we are not dealing with new firms, but existing companies. Therefore, the information that is used at the beginning of the period will provide similar variation across the years of the panel (see the summary statistics, Table 1 for the cross-sectional data and S7 Appendix for the full panel). Also, a cross-sectional analysis is suitable for the relationship between governance and risk because the governance index typically changes slowly from year to year within a company, whilst the variation between firms is substantial [53]. Third, the initial conditions (the data at the beginning of the period) do not provide feedback on future events and any potential endogeneity due to the feedback on some firm-level information with the growth measures is avoided.

**Table 1:** Descriptive statistics.

Variable	Observations	Mean	Std. Dev.	Min	Max	Skew	Kurt
***Risk variable***							
**RISK1**	760	0.061	0.064	0.004	0.269	2.038	6.593
**RISK2**	760	0.065	0.074	0.003	0.329	2.328	8.070
**RISK3**	760	0.063	0.070	0.003	0.310	2.284	7.867
**RISK4**	760	0.066	0.074	0.003	0.329	2.314	8.004
***Investor protection variables***							
**Corporate governance index**	760	0.536	0.120	0.244	0.829	-0.023	2.264
**Rule of law**	760	0.916	0.020	0.850	0.995	2.143	10.146
**Investor protection index**	760	0.574	0.182	0.000	0.700	-2.562	8.159
**Company independence**	760	0.560	0.497	0.000	1.000	-0.238	1.056
***Control variables***							
**ROA**	760	0.099	0.123	-0.304	0.269	-1.659	6.315
**Leverage**	760	0.220	0.176	0.003	0.624	0.749	2.721
**Size**	760	19.02	2.749	14.116	23.536	-0.158	2.028
***Growth (outcome) variables***							
**Sales growth**	760	0.105	0.131	-0.292	0.716	1.404	6.442
**Total assets growth**	760	0.103	0.135	-0.181	0.764	1.615	8.143

The numbers are not presented as percentages.

First, companies may choose to improve their corporate governance because they need more external financing, to increase their credibility and, therefore, profitability. Second, companies may choose to invest more in corporate governance to reduce the corporate risk. To mitigate this concern regarding reverse causality between the risk measure and the index of corporate governance, the firm-level corporate governance index and the control variables are measured at the beginning of our observation period– 2004. The use of the initial values of corporate governance index and control variables alleviates the concern that variation in the investment risk may cause the variation on corporate governance index.

As a benchmark model for the investment risk, we estimate Eq (1) using Ordinary Least Squares (OLS) model with industry fixed effects and allow for clustering of standard errors at the country level:

$${\text{Risk}}_{\text{i},\text{j},\text{k}\;(2004-2009)}=\beta_{0}+\beta_{1}{\text{FirmGov}}_{\text{i},\text{j},2004}+\beta_{2}{{\text{FirmGov}}^{2}}_{\text{i},\text{j},2004}+\beta_{3}{\text{CountryGov}}_{\text{j},2004}++\;\beta_{4}{\text{FirmControl}}_{\text{i},\text{j},2004}+\;\varphi_{k}\;+\varepsilon_{i,j,k}$$ (1)

where Risk_i,j,(2004–2009)_ is the *Risk* of firm *i* from country *j* and industry *k*, measured using the standard deviation of returns on assets for each firm over the period 2004–2009; FirmGov_i,j,2004_ represents the *Corporate governance index* of firm *i*, in country j and year 2004; CountryGov_j,2004_ is a vector of country-level governance control variables (*Strength of investor protection index* and *Rule of law*); FirmControl_i,j,2004_ is a vector of firm-level control variables (*Firm size*, *Return on assets*, *Leverage* and *Company independence)*; *φ*_*k*_ is a industry-specific effect, *ε*_*i*,*j*,*k*_ is the error term.

As robustness checks, we consider several extensions to the above model. First we test if the benchmark model is sensitive to functional form specifications by estimating a fractional logit and a fractional probit model, given that the *Risk* variable took values between 0 and 1. The results obtained with the fractional probability models are not statistically different than the results obtained using the linear model.

Second, we constructed a time varying risk measure by using a three-year rolling window. This time varying risk measure allows us to use the time varying information from the data and therefore, we can test if time varying information at the firm level is important and also if there are sources of simultaneity that link corporate governance to investment risk that need to be considered. Consequently, we estimate the model using panel data and GMM (using lags of governance as instruments for current governance) specifications:

$${\text{Risk}}_{\text{i},\text{j},\text{k},\text{j}}=\beta_{0}+\beta_{1}{\text{FirmGov}}_{\text{i},\text{j},\text{t}}+\beta_{2}{{\text{FirmGov}}^{2}}_{\text{i},\text{j},\text{t}}+\beta_{3}{\text{CountryGov}}_{\text{j},\text{t}}+\beta_{4}{\text{FirmControl}}_{\text{i},\text{j},\text{t}}+\;+\;\varphi_{k}\;+\varepsilon_{i,j,k,t.}$$ (2)

The results obtained using both panel data and GMM do not deviate from the OLS results, suggesting that indeed the benchmark specification estimates the relationship between corporate governance and investment risk assumed by a firm is consistently estimated and is robust to all these potential problems.

A second test that we implemented in our analysis is to see the effect of firm’s investment risk on firm’s growth. To assess this impact motivated by the previous discussion, we are using the following cross-sectional model:

$${Growth}_{\text{i},\text{j},\text{k},(2004-2009)}^{A/S}=\beta_{0}+\beta_{1}{RISK}_{i,j,k,(2004-2009)}+\beta_{2}{\text{CountryGov}}_{\text{j},2004}+\beta_{3}{\text{FirmControl}}_{\text{i},\text{j},2004}+\;\varphi_{k}\;+\varepsilon_{i,j,k}$$ (3)

where ${Growth}_{\text{i},\text{j},\text{k},(2004-2009)}^{A/S}$ represents the alternative measures of firm growth (average growth rate of total assets and average growth rate of sales) for the period 2004–2009.

To address potential endogeneity issues between company growth and risk, we have considered an instrumental-variables analysis using the IV 2SLS method. We employ the IV 2SLS method and use a set of instruments correlated with the firms’ risk measures but uncorrelated with the growth indicators. In line with the literature on corporate governance [11, 54, 55] we use as instruments the initial conditions (value at beginning of the observation period—2004) of the Risk variable. In the second model, the *Risk* variable is instrumented with the corporate governance index and its squared value, but also with the firm’s initial size. The choice of these instruments is motivated by: *i*) *Risk* measure has an inverted U-shaped relationship with the *Corporate governance index* (see in S2 Appendix), which means that the *Risk* is a function of both the *Corporate governance index* and its squared value and that between Risk and *Corporate governance index* is a nonlinear relationship; *ii*) the Risk measure is correlated with the *Corporate governance index* and *Corporate governance index* squared value, while Total Assets Growth and Sales Growth are not correlated with the *Corporate governance index* and *Corporate governance index* squared measures (see in S3 Appendix).

Using the information from the rolling window for the projected risk measure, a panel data growth measure is also estimated as a robustness check:

$${Growth}_{\text{i},\text{j},\text{k},\text{t}}^{A/S}=\beta_{0}+\beta_{1}{RISK}_{i,j,k,t}+\beta_{2}{\text{CountryGov}}_{\text{j},\text{t}}+\beta_{3}{\text{FirmControl}}_{\text{i},\text{j},\text{t}}+\;\varphi_{k}\;+\varepsilon_{i,j,k,t}$$ (4)

Additionally, to check if the Growth models (for Average Growth of Total Assets and Average Growth of Sales) are correlated, we use a Seemingly Unrelated Regression (SUR) bivariate model in which both equations for growth are estimated jointly:

{Growthi,j,k(2004−2009)A=β0+β1RISKi,j,k,(2004−2009)+β2CountryGovj,2004+β3FirmControli,j,2004+εi,j,kGrowthi,j,k(2004−2009)S=β0+β1RISKi,j,k,(2004−2009)+β2CountryGovj,2004+β3FirmControli,j,2004+εi,j,k (5)

In this case we check if a shock in one of the growth measures affects the other growth measure. The Seemingly Unrelated Regression (SUR) model is a generalization of a linear regression model that consists of several regression equations. SUR method assumes that all of the explanatory variables in each equation are exogenous [56].

## 4. Results

### 4.1. Data

Table 1 presents the descriptive statistics for the variables used in the analysis. The average of the corporate risk variable, reported in the first row, is about 6.1%, and varies between 0.4% and 27% for the companies in our sample. The value of investor protection is closed to the corporate governance index, with an average of 0.57 and a standard deviation higher than of the corporate governance index, meaning that the investor protection deviation between countries is higher than the corporate governance index across companies. In our sample approximately 56% of the companies are not dependent on large shareholders (none of the shareholders is owning more that 25% of the company). The mean of the return on assets variable is about 8.8%, with a standard deviation of 23.7%. The mean value of leverage is 22%, whilst the both growth variables have similar average values of about 10%.

Most variables (dependent, of interest and control) in our analysis are skewed and some have fatter tails. The two-stage procedure, which estimates a conditional mean effect, is not biased by the fact that these variables are not normally distributed. In fact, it is very hard to find normally distributed variables. The skewness of our dependent variables may, however, induce heteroscedasticity, which affects the estimation of the standard errors. For this reason, we use robust standard errors in our estimation.

S7 Appendix provides also summary statistics for the full panel. We observe that the average results at the cross-sectional level are very close to the average results on the full panel, which suggests a high persistency in the data. Also, these results may suggest that the estimated results obtained using the cross-sectional data and the panel data may not be significantly different.

To analyze the effect of corporate governance on firm risk, the sample is split in four groups (see S4 Appendix). Since the average risk value of companies with a lower (than median) level of corporate governance is higher than that of companies with a higher (above median) level (0.059 versus 0.062), this suggests that corporate governance may have a non-linear effect on the risk. This hypothesis will be tested in our model specification for the risk.

### 4.2. Analysis of the results

We proceed with the analysis following the steps described in the methodology section. First, we start by assessing the role of corporate governance in firm’s investment risk by estimating a cross-sectional linear model as a benchmark and as robustness checks, we look at functional form effects, time-varying and potential endogeneity effects, and second we link this analysis to understand the role of firm’s risk on firm’s growth.

#### 4.2.1. Firm-level risk

We report in Table 2 the determinants of the firm-level risk indicator (based on the benchmark risk measure *RISK1*), using the ordinary least squares (OLS) estimation framework as the base model, and Fractional Probit, Fractional Logit, GMM and Panel between estimator as robustness checks (based on *rolling SD*). To determine the impact of corporate governance on firm risk, the proxies of risk are regressed on firm-level and country-level corporate governance indices. Other determinants serve as control variables of risk.

**Table 2:** Marginal effect linear model, fractional probit, fractional logit and panel data between estimators.

Dependent variable: RISK1	(1)	(2)	(3)	(4)	(5)
	OLS—RISK1	ME fractional Probit RISK1	ME fractional Logit RISK1	GMM-RISK1	Panel data between estimator—Rolling SD
Corporate governance index	0.360***	0.370**	0.378***	0.333***	0.417***
	(0.131)	(0.147)	(0.144)	(0.070)	(0.107)
Corporate governance index^2^	-0.295**	-0.314**	-0.329**	-0.272***	-0.338***
	(0.121)	(0.135)	(0.134)	(0.056)	(0.092)
Company independence	-0.009***	-0.009*	-0.009*	-0.010***	-0.009*
	(0.004)	(0.004)	(0.004)	(0.001)	(0.002)
Investor protection	0.028**	0.027**	0.028**	0.047***	0.016**
	(0.011)	(0.011)	(0.012)	(0.006)	(0.007)
Rule of law	-0.523***	-0.482***	-0.489***	-0.268***	-0.310***
	(0.113)	(0.113)	(0.117)	(0.044)	(0.067)
Size	-0.0095***	-0.008***	-0.008***	-0.006***	-0.005***
	(0.004)	(0.001)	(0.001)	(0.001)	(0.001)
ROA	-0.098***	-0.049***	-0.045***	-0.049***	-0.037***
	(0.008)	(0.014)	(0.011)	(0.016)	(0.03)
Leverage	-0.024**	-0.023*	-0.025**	-0.029***	-0.024**
	(0.011)	(0.013)	(0.012)	(0.005)	(0.008)
Constant	0.614***			0.324***	
	(0.118)			(0.047)	
Observations	760	760	760	4560	4560
R-squared	0.307				
Turning Point (Inverse U-shape)	0.61	0.56	0.58	0.61	0.61
Industry FE	Yes	Yes	Yes	Yes	Yes

Robust standard errors in parentheses.

***, **, and * indicate significance at the 1%, 5%, and 10% levels. To test the inverted U-shape effect of Corporate Governance on firm’s risk we use a t-test based approach as in [59]. The Null Hypothesis in this case is: H0: Monotone or U shape versus the alternative H1: Inverse U shape was performed. The test statistic t-value = 3.89 and a p-value = 0.000, which suggest rejecting the Null Hypothesis in favor of the alternative. The 95% confidence interval for this turning point using a Fieller based method is [0.56; 0.66]. The validity of this test was proved [60].

To test our initial hypothesis that links corporate governance to firm’s risk, we add nonlinearities of governance measure in all model specifications. The results of all models (1–5) reveal a non-linear relationship between corporate governance indicators and firms`investment risk, as coefficients of both variables (the corporate governance index and its squared value) are statistically significant. This non-linear relationship could be motivated by several reasons. First, companies that want to maintain a higher standard of internal governance may incur additional costs. Therefore, designing an optimal governance model may require a careful consideration of all these factors that could have an impact on investment choices and corporate growth. Second, attempting to protect shareholder interests through further measures of compliance may introduce further operating complexities for management while increasing costs and reducing decision speeds and flexibility. The impact on firms forced to compete under such conditions will be considerable, particularly if they find themselves on an international landscape competing against firms not burdened with the same regulatory requirements.

We find a turning point value around 0.61 for the corporate governance index. The investment risk is increasing up to this level of corporate governance, after which higher levels of corporate governance are associated with a decrease in the investment risk. This level of internal corporate governance of 0.61 is higher than the average found in the sample (and also higher than average value of 0.58 of the US companies’ corporate governance index) and corresponds to companies with higher standards of corporate governance. In [52] it is argued that decision speed, flexibility, and innovation have often been viewed as key ingredients to business success. The increasing emphasis on legal and regulatory compliance speed up the to the collapse of WorldCom and Enron due to decisions that burdened management. These roadblocks in decision-making process are working against to protecting shareholders’ interests. Arguments in favor of increasing legal compliance are debated and the impacts of proposed regulatory compliance issues are discussed within the context of firm’s competitiveness and its need for speed and flexibility in decisions. The issue of increasing and stricter compliance for business is far-reaching.

Further, the results show that the degree of independence of a company with respect to its shareholder is negative and statistically significant. This is in line with [57] and [45] findings, showing that higher ownership concentration is associated with higher idiosyncratic risk. In our case the independent companies have lower corporate investment risk, comparing with firms that have dominant shareholders. Strength of investor protection index is positive and significant related to risk measures in all specifications. These results provide evidence that, as investor protection improves, there is less concern of expropriation by insiders, in the same time the controlling shareholders could not have an influence on decisions making process by managers to act according to their personal interests, making managers more or less risk averse [29, 11]. As most of companies are from countries with strong investor protection this results reflect that strong investor protection leads to higher risk-taking in investment.

Coefficients of the Rule of law proxy are negative and statistically significant. The results provide evidence that strengthening the rule of law can provide companies more certainty to invest and grow. Strong rule of law promotes a favorable environment for economic activity thought ensuring accountability, eliminating corruption, and providing certainty with respect to business transactions, fairness and transparency regarding litigations. In this way businesses are more focused on growing through its core activities, investing more in projects that can be acceptable risky, but in the same time value—enhancing. In fact, through better rule of law companies will improve long-term profitability and sustainability and will not face with higher corporate risk-taking. [11] find that the rule of law has no impact on the risk-taking proxy.

Control variables have in generally the expected sign. ROA has a negative and statistically significant coefficient, indicating that a higher return on assets is associated with a lower volatility of returns. The results are in line with the findings of [11]. Leverage is negatively associated with risk, with significance at 5% level. The negative sign and significant impact associated with leverage suggest that more leveraged companies are facing an increased level of being credit constrained by banks, and, consequently, they are trying to avoid high-risky projects, in order to pay for their obligations [11]. [58], while studying the same topic from both a macro and a microeconomic perspective, argued that higher leverage is a mechanism that imposes discipline on managers improving the efficiency of their decisions and their willingness to work harder to achieve profit-maximizing objectives. At the same time, higher leverage may increase the risk of bankruptcy. Our results are in contrast with the findings of [44] and [45] that showed a strong and positive relationship between leverage and the volatility of ROA and stock returns. [46], on the other hand, finds that the leverage has no impact on the return volatility for U.S. companies.

Similar to [61] and [11], our results reveal a negative and statistically significant relationship between the company size and corporate risk. This suggests that large companies have less exposure to risk, which is probably due to their ability to diversify their risk through different business lines, and also due to lower operating risks. In contrast, findings of [44] suggest that the volatility of return on assets is not significantly related to firm size.

Table 3 reports the determinants of the risk at firm-level, for several risk measures (RISK2, RISK3, RISK4), in addition to the benchmark measure (RISK1) reported previously.

**Table 3:** Robustness checks using alternative risk measures.

Dependent variable:	(1)	(2)	(3)	(4)
	RISK1	RISK2	RISK3	RISK4
Corporate governance index	0.360***	0.381**	0.374***	0.385***
	(0.131)	(0.153)	(0.142)	(0.154)
Corporate governance index^2^	-0.295**	-0.298**	-0.299**	-0.301**
	(0.121)	(0.141)	(0.131)	(0.141)
Company independence	-0.009***	-0.010**	-0.010**	-0.010**
	(0.004)	(0.005)	(0.004)	(0.005)
Investor protection	0.028**	0.013	0.031**	0.012**
	(0.011)	(0.013)	(0.012)	(0.013)
Rule of law	-0.523***	-0.569***	-0.556***	-0.570***
	(0.113)	(0.132)	(0.123)	(0.133)
Size	-0.0095***	-0.009***	-0.009***	-0.009***
	(0.004)	(0.001)	(0.001)	(0.001)
ROA	-0.098***	-0.123***	-0.115***	-0.123***
	(0.008)	(0.009)	(0.009)	(0.009)
Leverage	-0.024**	-0.024*	-0.025**	-0.023**
	(0.011)	(0.011)	(0.0123)	(0.013)
CONSTANT	0.614***	0.671***	0.656***	0.671***
	(0.118)	(0.138)	(0.128)	(0.138)
Observations	760	760	760	760
R-squared	0.307	0.300	0.324	
Industry FE	Yes	Yes	Yes	Yes

Robust standard errors in parentheses.

***, **, and * indicate significance at the 1%, 5%, and 10% levels.

The results using these alternative specifications of risk as robustness checks are similar with the results obtained for the benchmark measure of risk. The three alternative measures are very close with the 45-degree line (see S5 Appendix), which means that all four predictions of risk are very close to each other. This means that the predicted risk is stable across all four specifications and therefore, in the second stage we can use only the prediction of risk based on benchmark risk measure.

As some concerns may arise regarding the countries considered in our sample (e.g., Italy, UK, Japan are missing), we checked the robustness of our base model results by running the estimations on a reduced sample. We dropped Australia and South European countries and report the estimation results in S6 Appendix. The results of Model 1 (reduced sample) are similar comparing to Model 2 (benchmark sample).

To conclude, the results with alternative risk measures and with a reduced sample are robust compared to those obtained with the benchmark risk measure. Our findings support the hypothesis that corporate governance has an inverted U-shape effect on corporate risk.

#### 4.2.2. Risk and corporate growth

Table 4 exhibits the results of the impact of the firm-level risk measures on the growth of assets and sales at firm level.

**Table 4:** Firm-level growth 2SLS regressions—**Part 1**: First-Stage Regression. Firm-level growth 2SLS regressions—**Part 2**: Second-Stage Regressions of Company Growth Measures on RISK1.

VARIABLES	Company Sales Growth		Company Assets Growth	
	(1)	(2)	(3)	(4)
	RISK1	RISK1	RISK1	RISK1
*Instruments*				
Size_2004_	-0.005***	-0.009***	-0.005***	-0.005***
	(0.0007)	(0.358)	(0.000)	(0.000)
Corporate governance index_2004_		0.358***		0.0164**
		(0.131)		(0.006)
Corporate governance index^2^_2004_		-0.290**		-0.290**
		(0.121)		(0.121)
*Predetermined variables*				
ROA_2004_	-0.102***	-0.098***	-0.103***	-0.054***
	(0.009)	(0.009)	(0.008)	(0.015)
Leverage_2004_	-0.027**	-0.024**	-0.027**	-0.033***
	(0.011)	(0.011)	(0.011)	(0.004)
Company independence	-0.010**	-0.009**	-0.009**	-0.0122
	(0.004)	(0.004)	(0.005)	(0.0015)
Investor protection	-0.025**	-0.028**	0.025**	-0.033***
	(0.011)	(0.011)	(0.011)	(0.004)
Rule of Law	-0.340***	-0.523***	-0.340***	-0.0122
	(0.091)	(0.115)	(0.091)	(0.0015)
Constant	0.485***	0.614***	0.485***	0.165***
	(0.091)	(0.118)	(0.091)	(0.005)
R-squared	0.25	0.30	0.26	0.30
F value	45.96***	39.82***	48.28***	41.62***
Predictive power of Excluded Instruments				
F value	52.438***	29.034***	52.58***	29.14***
Partial R2	0.06	0.104	0.06	0.104
Instrumented Risk	1.223***	1.476***	2.029***	1.999***
	(3.66)	(5.35)	(0.396)	(0.311)
Company independence	0.004	0.007	0.012	0.011
	(0.41)	(0.64)	(0.013)	(0.013)
Investor protection	-0.091***	-0.092**	-0.137***	-0.131***
	(-3.37)	(-3.20)	(0.032)	(0.032)
Rule of Law	-0.531**	-0.726**	-0.405	-0.593***
	(-2.68)	(-2.84)	(0.235)	(0.289)
ROA_2004_	0.043	0.076*	0.257	0.258***
	(1.03)	(2.05)	(0.49)	(0.042)
Leverage_2004_	0.047	0.060	0.051	0.049
	(1.55)	(1.93)	(0.036)	(0.035)
Constant	0.555***	0.709**	0.391***	0.560***
	(2.94)	(2.96)	(0.224)	(0.270)
Observations	760	760	760	760
Hausman test (p-value)	30.264 (0.0)	20.107 (0.0)	25.81 (0.0)	42.43 (0.0)
Industry FE	Yes	Yes	Yes	Yes

Robust standard errors in parentheses,

*** significance for 1%,

** significance for 5%,

* significance for 10%

Since risk can be endogenous to firm growth, the ordinary least squares (OLS) method might provide inconsistent results. To address the endogeneity of investment risk choices with respect to company growth, we use two-stage least squares estimations. We instrument our Risk measure with the natural logarithm of total assets at the beginning of the observed period and also with the corporate governance index [11]. In addition, to capture the effect of the volatility of corporate governance, we extend the set of instrumental variables with the squared value of this index. Large companies are considered more stable from an operational point of view, which leads to less volatile returns. In contrast, a higher degree of risk is more likely specific to companies that operate in industries with higher growth rates.

The first- and second-stage regressions are presented in Table 4. We consider a linear model for the two-stage model, given that in the Risk model presented above there is not a significant difference between the results obtained using the OLS, Fractional Probit, Fractional Logit and GMM models. Consistent with our assumption about the association between instruments and risk, the first-stage estimations predict that risk is negatively related to the company size and positively/negatively associated with corporate governance. The instruments have an important predictive power given that partial-*R*^2^ of the first-stage regressions suggest that firm size explains between 6% and 10% of the variation in risk proxies. After performing a test of endogeneity, we reject the hypothesis that risk measure is exogenous and accept the alternative one. In addition, the F-test rejects the hypothesis, meaning that firm size and investor protection proxies are strong instruments. The second stage results provide evidence that there is a positive and statistically significant relationship between the instrumented proxy for the risk and both firm total assets and sales growth, meaning that higher volatility of return is associated with higher firm-level growth.

The results support our second hypothesis when we use instruments and control variable for independence of a company with respect to its shareholders, profitability and leverage. Part 1 of Table 4 shows the first-stage regressions of the models for the firm-level risk. The Part 2 of Table 4 presents the second stage results for the models of growth in sales and total assets. Models 1 and 3 are based on a single instrument (log of the initial size), whilst models 2 and 4 add to the instrument set the measures of corporate governance and its squared value.

The results of Model 1 from second-stage least square show that a one standard deviation increase in instrumented risk measure is associated with an increase of sales growth by 17.3%. Regarding assets, based on Model 3, a one standard deviation increase in risk would is associated with an increase in assets growth of 27%.

It is not clear whether or not the concentrated ownership will improve performance. First, providing better monitoring incentives to controlling shareholders could lead to better performance. In contrast, dominant shareholders could extract private benefits at the expense of minority shareholders. A higher level of ownership concentration indicates a stronger investor monitoring power regarding corporate managerial decision due to the incentives of investors to protect their investments. The proxy for independence of a company in our case is positive but not statistically significant, meaning that independent companies will grow faster, when comparing to companies with controlling shareholders. Return on assets is positive and statistically significant for Models 2 and 4, meaning that higher return on assets will leads to higher assets growth. Leverage has positive coefficients but is not statistically significant, while investor protection and rule of law have a negative impact on both growth measures.

As a conclusion, the results provide support for our second hypothesis, i.e. a positive relationship between risk instrumented and corporate governance and growth, which is consistent with the findings of [11].

Therefore, although it may be true that more risk in investment increases company growth in the U.S. and in countries with strong investor protection, this may not be the case in companies where risk is already relatively high. Once investment risk reaches a very high level then it is not clear whether more risk in investment will display higher growth and may actually work in the opposite direction. In fact, empirical studies suggest that at a low level of risk, growth increases as risk increases, but then declines as risk levels keep increasing. Risk sometimes is associated with corporate survival; for example, if companies will totally avoid investment risk, then they will become less competitive and consequently, will not survive.

In the last part of our analysis, we extend the model of [11] and consider that the two measures of interest (Total Assets and Sales) do not grow independently. Consequently, the two growth models are estimated jointly using a Seemingly unrelated regression (SUR) method, in which the Risk measure is replaced by a predicted Risk measure, obtained using the first stage regression of the previous analysis.

The results of the joint growth analysis, presented in Table 5, suggest that the two growth measures are not independent of each other (the Breusch-Pagan test of independence rejects independence). Moreover, the correlation between the errors in the two growth models is very high (0.71) and significant, which means that a positive shock to one of the growth measures affects the growth in the other measure in the same direction. Another interesting finding is that the effect on the predicted Risk measure is decreased in both equations when compared with the independently ran growth models.

**Table 5:** Seemingly Unrelated Regression (SUR).

VARIABLES	(1)	(2)
	Company sales growth	Company assets growth
Predicted Risk	2.080***	2.720***
	(0.339)	(0.347)
ROA	0.155***	0.348***
	(0.043)	(0.044)
Leverage	0.081**	0.0715*
	(0.029)	(0.030)
Company independence	0.014	0.0181
	(0.010)	(0.011)
Investor protection	-0.009	-0.075**
	(0.026)	(0.027)
Rule of Law	-1.480***	-1.581***
	(0.256)	(0.262)
CONSTANT	1.324***	1.373***
	(0.225)	(0.229)
Observations	760	760
R-squared	0.103	0.120

Robust standard errors in parentheses,

*** significance for 1%,

** significance for 5%,

* significance for 10%

#### 4.2.3. Robustness checks

As the initial period of analysis for all the firms in our data is 2004 (due to availability of other countries firm level data), as a robustness check we proceeded in two additional ways. First, we aggregated the time varying variables using a panel data between estimator to account for the average effect over the window of analysis for both variables of interest and control variables. Second, to consider the time varying information from the outcome variables and control variables, we also computed a time varying risk measure, by using a rolling window of three years. Subsequently we estimate a panel data model for both the new risk measure and the growth measures and results are presented in Table 6. The results obtained with both panel data between estimator used for the full window and with panel data based on the rolling window do not change in a significant way when compared to the cross-sectional model. We may conclude that our initial results are robust.

**Table 6:** Firm-level growth panel 2SLS regressions—**Part 1**: First-Stage Regression. **Part 2**: Second-Stage Regressions of Company Growth Measures on RISK1 and Rolling SD.

VARIABLES	Company Sales Growth		Company Assets Growth	
	(1)	(2)	(3)	(4)
	Rolling SD	SD(RISK1)	Rolling SD	SD (RISK1)
*Instruments*				
Size	-0.005***	-0.006***	-0.005***	-0.006***
	(0.0007)	(0.000)	(0.000)	(0.000)
Corporate governance index	0.414***	0.333***	0.402***	0.351**
	(0.106)	(0.143)	(0.106)	(0.143)
Corporate governance index^2^	-0.335***	-0.288**	-0.325***	-0.301**
	(0.091)	(0.125)	(0.091)	(0.126)
*Predetermined variables*				
ROA	-0.022***	-0.064***	-0.037***	-0.066***
	(0.002)	(0.004)	(0.002)	(0.004)
Leverage	-0.021***	-0.033***	-0.023***	-0.033***
	(0.007)	(0.010)	(0.007)	(0.010)
Company independence	-0.009***	-0.010***	-0.009***	-0.0109
	(0.002)	(0.004)	(0.003)	(0.003)
Investor protection	-0.022***	0.034***	0.018**	-0.032***
	(0.007)	(0.009)	(0.007)	(0.009)
Rule of Law	-0.304***	-0.313***	-0.299***	-0.324
	(0.068)	(0.092)	(0.068)	(0.093)
Constant	0.314***	0.382***	0.313***	0.392***
	(0.071)	(0.097)	(0.071)	(0.097)
R-squared	0.22	0.27	0.28	0.30
F value	39.77***	54.12***	56.08***	62.63***
Instrumented Risk	1.103***	1.088***	1.450***	1.532***
	(0.395)	(0.307)	(0.422)	(0.324)
Company independence	-0.022*	-0.010	-0.010	-0.005
	(0.01)	(0.011)	(0.012)	(0.013)
Investor protection	-0.052*	-0.075***	-0.137***	-0.149***
	(0.03)	(0.027)	(0.030)	(0.028)
Rule of Law	-1.080***	-0.684***	-0.770***	-0.467*
	(0.256)	(0.241)	(0.269)	(0.236)
ROA	0.021	0.028	0.067***	0.104***
	(0.013)	(0.025)	(0.021)	(0.027)
Leverage	-0.011	0.000	-0.020	0.070**
	(0.03)	(0.033)	(0.036)	(0.035)
Constant	1.100***	0.723***	0.827***	0.511**
	(0.24)	(0.227)	(0.253)	(0.247)
Observations	3040	4560	3040	4560
Nr of firm	760	760	760	760
Hausman test (p-value)	31.254 (0.0)	22.007 (0.0)	27.63 (0.0)	45.19 (0.0)
Industry FE	Yes	Yes	Yes	Yes

Robust standard errors in parentheses,

*** significance for 1%,

** significance for 5%,

* significance for 10%

Additionally, given the construction of our time varying risk measure, we are able to quantify the effect of the crisis both on our risk measure and also on our two growth measures. In order to do this we generated crisis dummies for 2008 and 2009 and added them in the two regressions.

From the results with crisis dummies, presented in Table 7, we observe that 2008 was an year with no effect for the risk and growth measures, while coefficients for year 2009 show a significant effect of the crisis on both risk measure (standard deviation of ROA and rolling window standard deviation) and the two growth measures—sales and total assets growth. This delayed effect of the financial crisis on the firm growth is justified: the financial crisis was transmitted instantaneously at financial institutions level, but not at non-financial firm level. In addition, we estimated a model where we interact crises dummy with the governance and squared governance. The results of this model with interaction presented in S8 Appendix also suggest that during crises the governance has also a nonlinear effect on risk.

**Table 7:** Firm-level growth panel 2SLS regressions with crises dummy—**Part 1**: First-Stage Regression. **Part 2**: Second-Stage Regressions of Company Growth Measures on Rolling SD with crises dummy.

VARIABLES	Company Sales Growth	Company Assets Growth
	(1)	(2)
	Rolling SD	Rolling SD
*Instruments*		
Size	-0.006***	-0.006***
	(0.0007)	(0.000)
Corporate governance index	0.416***	0.405***
	(0.106)	(0.106)
Corporate governance index^2^	-0.331***	-0.320***
	(0.091)	(0.091)
*Predetermined variables*		
ROA	-0.022***	-0.037***
	(0.002)	(0.002)
Leverage	-0.020***	-0.021***
	(0.007)	(0.007)
Company independence	-0.009***	-0.009***
	(0.002)	(0.003)
Investor protection	-0.028***	0.026**
	(0.008)	(0.008)
Rule of Law	-0.299***	-0.289***
	(0.068)	(0.068)
Year2008	0.006	-0.027
	(0.041)	(0.01)
Year2009	-0.135***	-0.05***
	(0.024)	(0.06)
Constant	0.313***	0.310***
	(0.071)	(0.071)
R-squared	0.22	0.29
F value	32.37***	45.85***
Instrumented Risk	1.455***	1.709***
	(0.389)	(0.416)
Company independence	-0.019	-0.008
	(0.012)	(0.012)
Investor protection	-0.015	-0.084**
	(0.03)	(0.033)
Rule of Law	-0.861***	-0.631**
	(0.259)	(0.274)
ROA	0.023*	0.075***
	(0.013)	(0.021)
Leverage	0.012	-0.004
	(0.03)	(0.036)
Year2008	0.006	-0.037
	(0.041)	(0.04)
Year2009	-0.135***	-0.07***
	(0.024)	(0.258)
Constant	0.880***	0.687***
	(0.24)	(0.258)
Observations	3040	3040
Nr of firm	670	670
Hausman test (p-value)	31.256 (0.0)	27.63 (0.0)
Industry FE	Yes	Yes

Robust standard errors in parentheses,

*** significance for 1%,

** significance for 5%,

* significance for 10%

## Conclusion

In this paper we use a large multi-country sample of non-financial companies for a period that includes one major episode of financial crisis and provide empirical evidence on the impact of corporate governance on company risk, but also on the moderating effect of corporate governance on the relation between firm-level risk and growth at firm level.

We document a non-linear inverted U-shape relationship between corporate governance and firms’ investment risk, with a turning point for the level of corporate governance index identified at 0.61. The decrease in investment risk at high levels of corporate governance is possible due to additional requirements and to the increase in costs needed to maintain higher standards of internal governance.

Furthermore, the results obtained from a two-stage regression support our second hypothesis regarding a positive and statistically significant relationship between risk and growth at firm level. This result is robust to other risk measures that capture deviations of the risk of assets from its average at country level, at industry level and jointly at country and industry level.

The results of our analysis confirm that quality of internal governance leads to higher firm-level riskiness in investment, which in turn is positively associated with company growth. Furthermore, we show that a shock to one of the growth measures (sales or assets) affects the other growth measure in the same direction very strongly.

Additionally, with the help of a new time varying risk measure, we quantify the effect of the crisis on both, our risk measure and also on our two growth measures. The results that control for crisis reveal that both risk and growth measures are affected in the second year of the crisis, with a significant effect identified for the growth on sales. The lag effect of the financial crisis on the firm growth is supported by the fact that the financial crisis was transmitted instantaneously at financial institutions level, but not at non-financial firm level.

This empirical research contributes to the extension of existing literature for two reasons. First, it employs a firm-level index to document the impact of corporate governance on investment risk for a period leading to crisis. Second, this paper is one of the firsts that emphasize the non-linear impact of the firm-level corporate governance on investment risk that further will lead to corporate growth.

Further research should consider extending the timespan of the analysis for the post financial crisis period, as dramatic changes in firms’ risk have taken place after this recent global shock.

## Supporting information

S1 Appendix Variable definitions. (DOCX)

S2 Appendix RISK1 measure as a function of governance index. (DOCX)

S3 Appendix Correlation matrix. (DOCX)

S4 Appendix Distribution of the risk measure by the quantiles of corporate governance index. (DOCX)

S5 Appendix Alternative predicted risk measures as a function of the benchmark predicted risk. (DOCX)

S6 Appendix Robustness check of the base specification by reducing the working sample. (DOCX)

S7 Appendix Descriptive statistics for panel data analysis. (DOCX)

S8 Appendix Linear model with crises interaction for risk model. (DOCX)
