# Supplementary material for: The nexus between corporate governance, risk taking, and growth
Source: PLoS One. 2020 Feb 4;15(2):e0228371. doi: 10.1371/journal.pone.0228371 (PMC6999870; doi:10.1371/journal.pone.0228371)
Supplement: S1 Appendix — (DOCX) [file pone.0228371.s001.docx]

**APPENDICES**

**Appendix A** *Variable definitions*

| **Variables** | **Definition** | **Source** |
| --- | --- | --- |
| *Risk variables* | | |
| Risk measures | For each company with available EBITA and Total Assets for at least 5 years, we compute ROA and deviation from company ROA. The standard deviation of this measure is then established as the value of risk.  ${\boldsymbol{RISK}\boldsymbol{1}}_{i}=\sqrt{\frac{1}{T-1}\sum_{t=2004}^{T} \left( {ROA}_{it}-\frac{1}{T}\sum_{t=1}^{T} {ROA}_{it}) \right)^{2},}t=2004\ldots2009$  Additional measures of standard deviation risks measures are created using deviations of ROAs from their:   - ${\boldsymbol{RISK}\boldsymbol{2}}_{\boldsymbol{i}} -$ using country means  (ROA*^c^_it_*=${ROA}_{itc}-\frac{1}{Nc}\sum_{c=1}^{Nc} {ROA}_{itc})$, where *i* represents the firm*, c* represents a specific country in the sample and $N$c is the number of countries. - ${\boldsymbol{RISK}\boldsymbol{3}}_{i} -$ using industry means  (ROA*_it_^I^* =${ROA}_{itI}-\frac{1}{Nind}\sum_{I=1}^{Nind} {ROA}_{itI})$, where *I* represents the industry at 1 digit SIC code and $Nind$ represents the number of industries at 1 digit SIC code. - ${\boldsymbol{RISK}\boldsymbol{4}}_{\boldsymbol{i}} -$ using country and industry means   (ROA*_it_^cI^*=${ROA}_{itcI}-\frac{1}{Nc\cdot Nind}\sum_{c=1}^{Nc} \sum_{I=1}^{Nind} {ROA}_{itcI})$.  **Rolling SD**- rolling window standard deviation of ROA, by using a three-year rolling window.  $\mathbf{ROLLING}\boldsymbol{SD}=\sqrt{\frac{1}{n-1}\sum_{j=t}^{t+n} \left( {ROA}_{ij}-\frac{1}{n}\sum_{j=t}^{t+n} {ROA}_{ij}) \right)^{2},}$  $n=3$, j=2004, 2005, 2006, 2007. | Orbis |
| *Investor protection variables* | | |
| Corporate governance index (GOV) | The index consists of 41 attributes common to both U.S. and non-U.S. firms that cover four large subcategories: Board (24 attributes), Audit (three attributes), Anti-takeover provisions (six attributes), and Compensation and ownership (eight attributes). If a firm satisfies all 41 governance attributes, then its GOV41 index will be equal to 1. | Index is from Aggarwal et al., 2011 |
| Strength of investor protection (IP) | The index is composed using the average of three indicators, respectively the extent of disclosure index, the extent of director liability index, and the ease of shareholder suit index. The index takes value from 0 to 1, with higher values attributed to better investor protection. | World Bank |
| Rule of law (RL) | Rule of law indicates the effectiveness of regulatory enforcement. This index takes value from 0 to 1, with higher values attributed to better rule of law. | World Bank |
| *Firm level control variables* | | |
| Initial firm size (SIZE) | Defined as the natural logarithm of total assets. The variable has been retrieve as of the beginning of my observation period, respectively 2002. | Orbis |
| Initial Leverage (LEV) | Defined as the ratio of the long-term debt to total assets. The variable has been retrieved as of the beginning of my observation period, respectively 2002. | Orbis |
| Initial return on assets (ROA) | Defined as the ratio of EBITDA to total assets. The variable has been retrieve as of the beginning of my observation period, respectively 2002. | Orbis |
| Company independence (INDEP) | To characterize the degree of independence of a corporation with respect to its shareholders, we use the BvD Independence Indicator. For computational reasons, the BvD Independence Indicator has been transformed into a dummy variable taking values of 1 if the company is rated with indicator A, attaching to any company with known recorded shareholders none of which having more than 25% of direct or total ownership, and zero otherwise. | Orbis |
| Sales growth (SALES_GR) | Firm level growth is measured as the average of the growth in sales over the sample period, respectively 2002-2009. | Orbis |
| Assets growth (TA_GR) | Firm level growth is measured as the average of the growth in total assets, over the sample period, respectively 2002-2009. | Orbis |
