# Supplementary figures and images for: The nexus between corporate governance, risk taking, and growth
Source: PLoS One. 2020 Feb 4;15(2):e0228371. doi: 10.1371/journal.pone.0228371 (PMC6999870; doi:10.1371/journal.pone.0228371)

**APPENDIX B**

*RISK1 measure as a function of Governance Index*


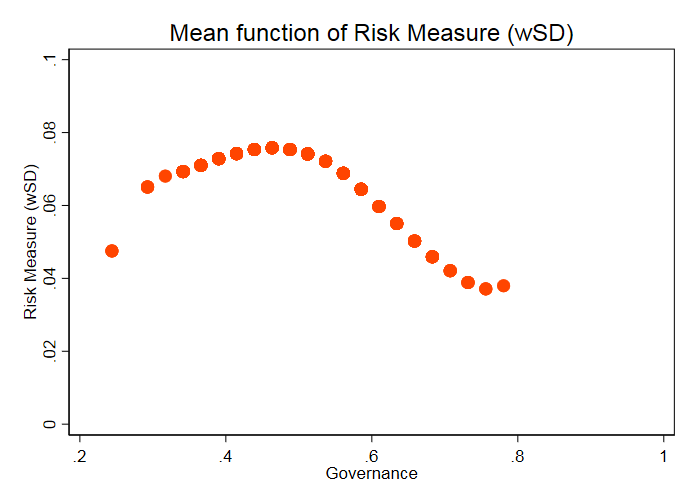

Supplement: S2 Appendix — (DOCX) [file pone.0228371.s002.docx]

**APPENDIX E**

*Alternative Predicted Risk Measures as a function of the Benchmark Predicted Risk*


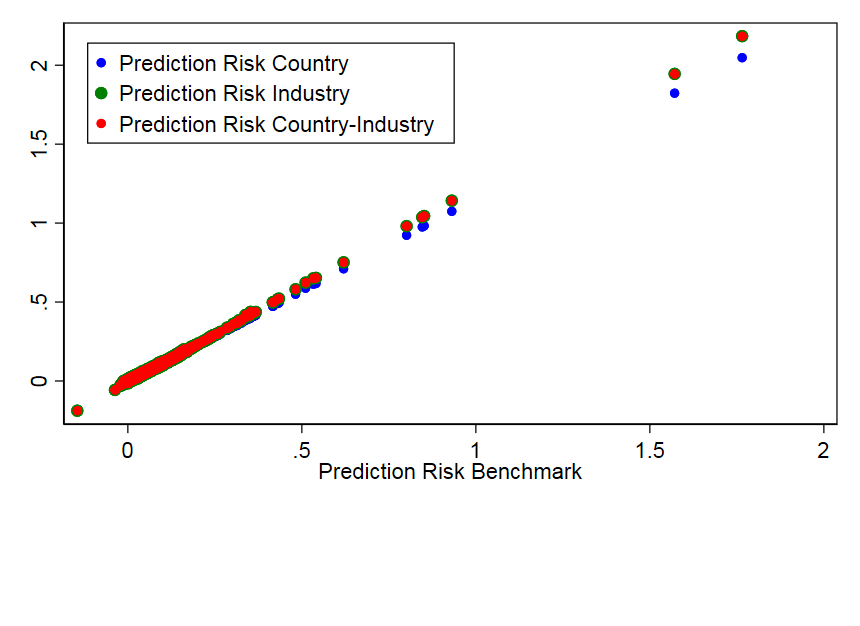

Supplement: S5 Appendix — (DOCX) [file pone.0228371.s005.docx]
