# Supplementary material for: The nexus between corporate governance, risk taking, and growth
Source: PLoS One. 2020 Feb 4;15(2):e0228371. doi: 10.1371/journal.pone.0228371 (PMC6999870; doi:10.1371/journal.pone.0228371)
Supplement: S3 Appendix — (DOCX) [file pone.0228371.s003.docx]

**APPENDIX C**

*Correlation matrix*

| Correlation coefficient | Risk | Total Assets | Sales |
| --- | --- | --- | --- |
| Size | -0.3648 | -0.0812 | -0.0412 |
| Corporate governance | -0.1502 | 0.0303 | 0.0486 |
| Corporate governance squared | -0.1630 | 0.0229 | 0.0334 |
