# Supplementary material for: The nexus between corporate governance, risk taking, and growth
Source: PLoS One. 2020 Feb 4;15(2):e0228371. doi: 10.1371/journal.pone.0228371 (PMC6999870; doi:10.1371/journal.pone.0228371)
Supplement: S4 Appendix — (DOCX) [file pone.0228371.s004.docx]

**APPENDIX D**

*Distribution of the Risk measure by the quantiles of Corporate Governance Index*

| Variables | Corporate Governance Centile | | | |
| --- | --- | --- | --- | --- |
|  | <25 | 25-50 | 50-75 | >75 |
| RISK1 | 0.0617 | 0.0628 | 0.0702 | 0.051 |
