# Supplementary material for: The nexus between corporate governance, risk taking, and growth
Source: PLoS One. 2020 Feb 4;15(2):e0228371. doi: 10.1371/journal.pone.0228371 (PMC6999870; doi:10.1371/journal.pone.0228371)
Supplement: S6 Appendix — (DOCX) [file pone.0228371.s006.docx]

**APPENDIX F**

*Robustness check of the base specification by reducing the working sample*

|  | (1) | (2) |
| --- | --- | --- |
| VARIABLES | RISK1 | RISK1 |
|  |  |  |
| Corporate governance index | 0.289** | 0.360*** |
|  | (0.139) | (0.132) |
| Corporate governance index^2^ | -0.220* | -0.291** |
|  | (0.127) | (0.121) |
| Company independence | -0.00918** | -0.00953** |
|  | (0.00434) | (0.00423) |
| Investors protection | 0.0315*** | 0.0287** |
|  | (0.0115) | (0.0114) |
| Rule of Law | -0.775*** | -0.523*** |
|  | (0.133) | (0.114) |
| Size | -0.0116*** | 0.00929*** |
|  | (0.00123) | (0.00115) |
| ROA | -0.0926*** | -0.0982*** |
|  | (0.00856) | (0.00851) |
| Leverage | -0.0175 | -0.0242** |
|  | (0.0115) | (0.0114) |
| Constant | 0.904*** | 0.614*** |
|  | (0.140) | (0.118) |
| Observations | 723 | 760 |
| R-squared | 0.337 | 0.307 |
| Standard errors in parentheses, *** p<0.01, ** p<0.05, * p<0.1 | | |
| Note: Model 1 - Reduced sample, without Australia and Southern European countries; Model 2 - Base sample. | | |
