# Supplementary material for: The nexus between corporate governance, risk taking, and growth
Source: PLoS One. 2020 Feb 4;15(2):e0228371. doi: 10.1371/journal.pone.0228371 (PMC6999870; doi:10.1371/journal.pone.0228371)
Supplement: S7 Appendix — (DOCX) [file pone.0228371.s007.docx]

**APPENDIX G**

*Descriptive statistics for panel data analysis*

| **Variable** | **Observations** | | **Mean** | | **Std. Dev.** | | **Min** | | **Max** | |
| --- | --- | --- | --- | --- | --- | --- | --- | --- | --- | --- |
| ***Risk variable*** | | | | | | | | | | |
| **Rolling SD** | | 3040 | | 0.059 | | 0.162 | | 0.0002 | | 0.423 |
| ***Investor protection variables*** | | | | | | | | | | |
| **Corporate governance index** | | 3040 | | 0.583 | | 0.120 | | 0.219 | | 0.902 |
| **Rule of law** | | 3040 | | 0.917 | | 0.018 | | 0.840 | | 1.000 |
| **Investor protection index** | | 3040 | | 0.593 | | 0.159 | | 0.000 | | 0.700 |
| **Company independence** | | 3040 | | 0.592 | | 0.491 | | 0.000 | | 1.000 |
| ***Control variables*** | | | | | | | | | | |
| **ROA** | | 3040 | | 0.077 | | 0.127 | | -0.304 | | 0.269 |
| **Leverage** | | 3040 | | 0.203 | | 0.179 | | 0.003 | | 0.624 |
| **Size** | | 3040 | | 19.244 | | 2.599 | | 14.116 | | 23.536 |
| ***Growth (outcome) variables*** | | | | | | | | | | |
| **Yearly sales growth** | | 3040 | | 0.113 | | 0.236 | | -0.292 | | 0.716 |
| **Yearly total assets growth** | | 3040 | | 0.108 | | 0.230 | | -0.239 | | 0.764 |
