# Supplementary material for: The nexus between corporate governance, risk taking, and growth
Source: PLoS One. 2020 Feb 4;15(2):e0228371. doi: 10.1371/journal.pone.0228371 (PMC6999870; doi:10.1371/journal.pone.0228371)
Supplement: S8 Appendix — (DOCX) [file pone.0228371.s008.docx]

**APPENDIX H**

*Linear Model with crises interaction for risk model*

|  | (1) |
| --- | --- |
| VARIABLES | OLS-RISK1 |
|  |  |
| Corporate governance index | 0.215*** |
|  | (0.053) |
| Corporate governance index^2^ | -0.189*** |
|  | (0.047) |
| Corporate governance index*Crises | 0.293*** |
|  | (0.113) |
| Corporate governance index^2^*Crises | -0.194** |
|  | 0.094 |
| Size | -0.006*** |
|  | (0.01) |
| Company independence | -0.012*** |
|  | (0.001) |
| Investor protection | 0.365*** |
|  | (0.005) |
| Rule of law | -0.235*** |
|  | (0.371) |
| ROA | -0.056*** |
|  | (0.002) |
| Leverage | -0.030*** |
|  | (0.01) |
| Constant | 0.339*** |
|  | (0.039) |
|  |  |
| Observations | 4560 |
| R-squared | 0.252 |
